# Changing Bee and Hoverfly Pollinator Assemblages along an Urban-Rural Gradient

**DOI:** 10.1371/journal.pone.0023459

**Published:** 2011-08-12

**Authors:** Adam J. Bates, Jon P. Sadler, Alison J. Fairbrass, Steven J. Falk, James D. Hale, Tom J. Matthews

**Affiliations:** 1 Geography, Earth and Environmental Sciences, The University of Birmingham, Birmingham, West Midlands, United Kingdom; 2 Warwickshire Museum, Warwick, Warwickshire, United Kingdom; Trinity College Dublin, Ireland

## Abstract

**Background:**

The potential for reduced pollination ecosystem service due to global declines of bees and other pollinators is cause for considerable concern. Habitat degradation, destruction and fragmentation due to agricultural intensification have historically been the main causes of this pollinator decline. However, despite increasing and accelerating levels of global urbanization, very little research has investigated the effects of urbanization on pollinator assemblages. We assessed changes in the diversity, abundance and species composition of bee and hoverfly pollinator assemblages in urban, suburban, and rural sites across a UK city.

**Methodology/Principal Findings:**

Bees and hoverflies were trapped and netted at 24 sites of similar habitat character (churchyards and cemeteries) that varied in position along a gradient of urbanization. Local habitat quality (altitude, shelter from wind, diversity and abundance of flowers), and the broader-scale degree of urbanization (e.g. percentage of built landscape and gardens within 100 m, 250 m, 500 m, 1 km, and 2.5 km of the site) were assessed for each study site. The diversity and abundance of pollinators were both significantly negatively associated with higher levels of urbanization. Assemblage composition changed along the urbanization gradient with some species positively associated with urban and suburban land-use, but more species negatively so. Pollinator assemblages were positively affected by good site habitat quality, in particular the availability of flowering plants.

**Conclusions/Significance:**

Our results show that urban areas can support diverse pollinator assemblages, but that this capacity is strongly affected by local habitat quality. Nonetheless, in both urban and suburban areas of the city the assemblages had fewer individuals and lower diversity than similar rural habitats. The unique development histories of different urban areas, and the difficulty of assessing mobile pollinator assemblages in just part of their range, mean that complementary studies in different cities and urban habitats are required to discover if these findings are more widely applicable.

## Introduction

Insects, especially bees, are thought to be the most important group of pollinators globally [1], [2], [3], [4], [5]. The recent well-documented declines in North America and Europe of the European Honeybee (*Apis melifera*) and other insect pollinators, sometimes termed the ‘pollination crisis’, has been the subject of considerable media, public, political, and academic interest [2], [3], [4], [6], [7]. Whether these declines will cause significant declines in crop and wild plant populations is the subject of some debate however. Most flowering plants are generalists in terms of their pollination requirements; so the decline or disappearance of one species will usually still leave other species available for the provision of pollination services [2], [8], [9], [10], [11]. In addition, many flowering plants are not directly reliant on pollinators for reproduction, as they are able to self-pollinate [1], [8]; although inbreeding depression and lower crop quality can result [2]. Nevertheless, an estimated 70% of the different types of world crop that are directly used for human consumption are dependent on animal-mediated pollination to some extent [1], and a conservative estimate of the value of this pollination service was €153 billion for 2005 [12]. There is also growing empirical evidence to support the proposition that the diversity of insect pollinator assemblages influences the reproduction and diversity of wild flowering plants [6], [13], of which 87.5% are estimated to be animal pollinated worldwide [14]. Although the significance of pollinator loss is debated in the literature, most authorities agree that this is an issue of global concern that warrants further research [2], [4], [8], [11], [15].

There has been a considerable amount of research on the response of insect pollinators to anthropogenic disturbances caused by agricultural intensification including, but not limited to; loss and fragmentation of natural habitat, loss of nesting resource, reduced floral diversity, and agro-chemical effects [2], [7], [16], [17]. There are some distinct differences between the nature of urbanized and agricultural landscapes such as the more heavily modified climate [18], and more spatial habitat diversity at smaller scales in urban, compared to agricultural areas. However, for the most part, urbanization is associated with similar anthropogenic disturbances [19], [20], [21], [22] characteristic of agricultural areas, which result in a loss of resources for insects and increased stress on populations.

As yet, relatively few studies have looked at the effect of urbanization on pollinator assemblages. This is perhaps unsurprising as the most direct ecosystem service delivered by pollinators is pollination of crops, and urban areas are traditionally considered of limited importance in this respect. There are, however, three distinct reasons why understanding the effects of urbanization on pollinator assemblages is important: (1) the intrinsic conservation value of urban pollinators, (2) the utilitarian ecosystem service value of urban pollinators, and (3) the use of urbanization gradients as space for time proxies of the future effects of continuing urbanization; each is briefly described. (1) Urban landscapes can support many species of significant local, and sometimes national, intrinsic conservation value [23], [24], [25]. There is little information on the conservation value of bees and hoverflies in urban areas, but pollinators will likely affect the prospects of flowering plants of conservation importance through their reciprocal dependence. (2) Through their influence on flowering plants, urban pollinators can influence a wide range of ecosystem services; including (*sensu* [26]): provisioning (e.g. food, materials), regulating (e.g. climate regulation, hydrology), supporting (e.g. primary production, soil formation), and cultural (e.g. aesthetic, education) ecosystem services. Their most apparent ecosystem service is the support of urban food production through pollination. The importance of garden and allotment food production in the developed world is poorly known. However, studies in the developing world, where urban food production is much more extensive, suggest that urban agriculture can provide extra nutrition and food security for households [27], [28]. (3) Landscapes are becoming increasingly urbanized. Currently around half the world's population live in urban areas and this is set to increase dramatically during the next 50 years [29]. The potentially far-reaching effects of urbanization on landscape pollination service are difficult to predict, especially when empirical data are limited.

Research on pollinators in urban areas has focused on various environments and assemblages and used a variety of study methods (see [30] for summary for bees). Few studies have sampled bees in comparable habitats across the whole urban-rural gradient (but see [31]), and no study to our knowledge has yet examined hoverflies. Pervasive patterns are the strong importance of local habitat quality and a shift in assemblage structure with urbanization [7], [31], [32], [33], but the effects of urbanization on pollinator abundance and diversity have varied.

Here, we use a gradient approach [34] to investigate the diversity, abundance and species assemblage composition of bees and hoverflies in urban, suburban and rural sites in and around the City of Birmingham in the UK. In particular it focuses on how these assemblages respond to local site quality and landscape measures of urbanization intensity. Unlike other studies we used detailed environmental data from a GIS to create an *a priori* gradient [35], which was then used as a tool for site selection. Further site based and landscape variables were then derived from the GIS and used to model the environment-pollinator assemblage variability across the pool of sites. The aim was to characterize changes in pollinator assemblages along an urbanization gradient. We addressed four specific objectives: (i) to characterize changes in pollinator diversity; (ii) to characterize changes in pollinator abundance, (iii) to characterize compositional changes of pollinator assemblages, and (iv) to investigate how objectives i–iii respond to changes in site quality and landscape indicators of urbanization intensity. We achieved our aims and objectives, but explore in the Discussion section why the findings of this investigation cannot be extrapolated to a general theory of pollinator response to urbanization until further studies investigate changes in pollinator assemblage in different cities in multiple habitat types.

## Results

**Figure 1:**
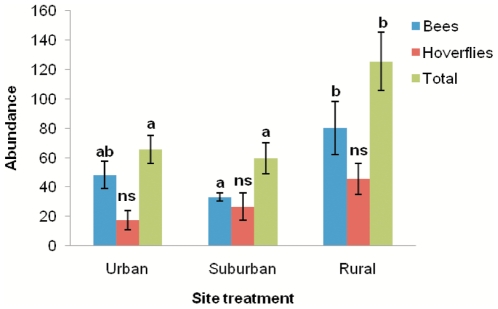
Relationships between site treatment and total abundance of bees, hoverflies and total pollinators. Error bars  = +/− 1SE. Bars that do not share a letter showed significant differences (*P*<0.05) between treatments. Ns  =  no significant difference.

**Figure 2:**
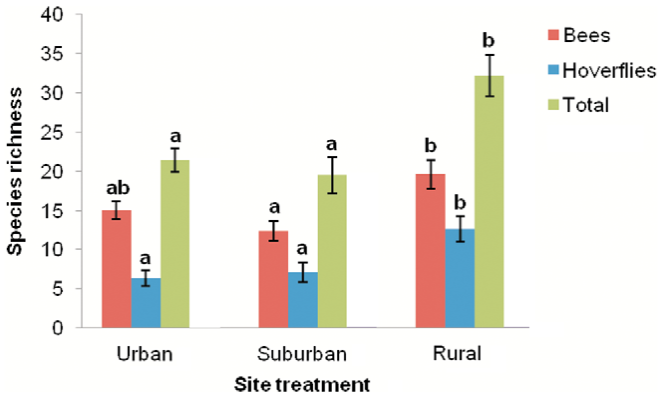
Relationships between site treatment and total species richness of bees, hoverflies, and total pollinators. Error bars  =  +/− 1SE. Bars that do not share a letter showed significant differences (*P*<0.05) between treatments.

A total of 1292 individuals of 58 species of bees and 714 individuals of 50 species of hoverfly were recorded in the study. The European Honeybee (*Apis melifera*) made up less than 5% of the total catch, and no more than 23% at any one site. The total abundance of bees, and total pollinators (bees plus hoverflies) were found to significantly differ between the urban, suburban, and rural treatments (Table 1). There was a significantly greater total abundance of bees at rural sites than at suburban sites, and significantly more total pollinators at rural sites than urban and suburban sites (Figure 1). The total species richness of bees, hoverflies and total pollinators were also significantly different between site types (Table 1), with a higher species richness of bees in rural sites than suburban sites; of hoverflies in rural sites than urban and suburban sites; and of total pollinators in rural sites than urban and suburban sites (Figure 2).

**Table 1:** Means tests for differences between Urban, Suburban, and Rural treatments.

	Degrees of freedom	Test statistics	Significance
**Bee abundance**	2	***H*** = 6.90*	**0.03180**
**Hoverfly abundance**	2	*F* = 2.61	0.09754
**Total abundance**	2	***H*** = 7.75*	**0.02081**
**Bee species richness**	2	***F*** = 6.26**	**0.00738**
**Hoverfly species richness**	2	***F*** = 6.64**	**0.00582**
**Total species richness**	2	***F*** = 9.84***	**0.00097**

*H* = Kruskal-Wallace non-parametric test statistic, *F* = ANOVA test statistic, significant values at *P* = 0.05 are highlighted in bold. Statistical significance at *P* = 0.05, 0.01, and 0.001 shown with *, **, and *** respectively.

**Figure 3:**
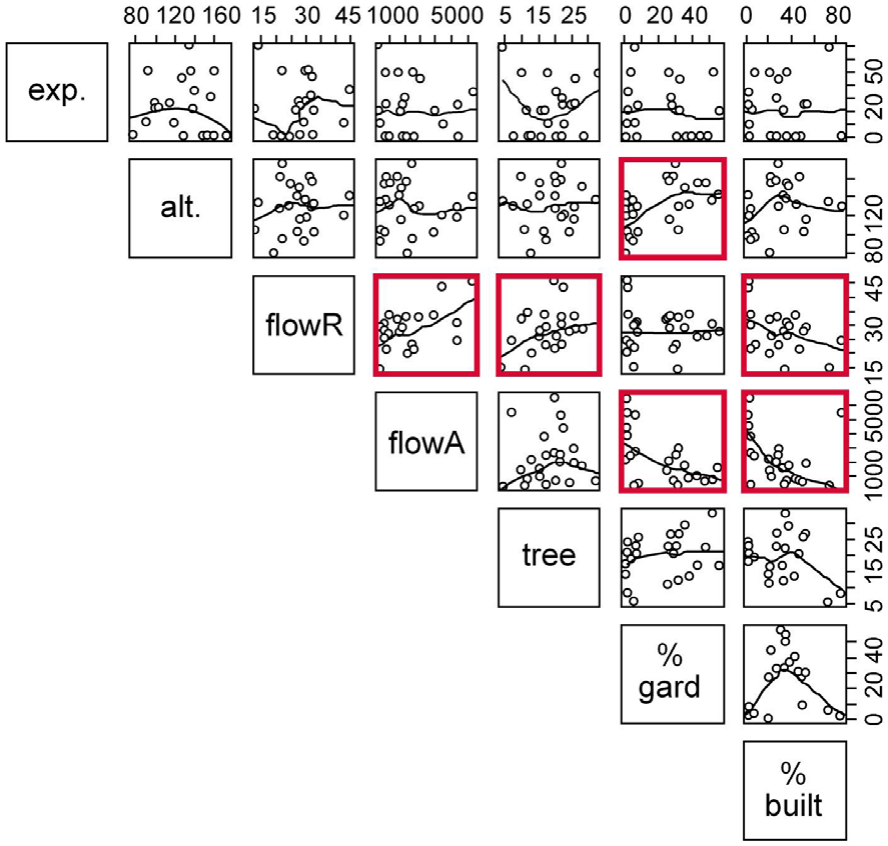
Co-plot showing relationships between explanatory variables used in the generalized linear models. Pairwise scatterplots are shown with locally weighted loess smoothing to aid visual interpretation and panels with a Pearson correlation >0.3 highlighted. Variable codes are abbreviated for clarity (exp.  = % exposure, alt.  =  altitude in metres, flowR  =  flowering forb species richness, flowA  =  flowering forb flower abundance, tree  =  number of flowering trees, % gard.  = % gardens within 500m, and% built  = % built space within 500m). Percentage gardens and built space at 500m scale was used as an example because this was most representative of these variables at all wider landscape scales.

Table 2 illustrates the method used for selecting the best GLM for each abundance and richness metric (in this case, for total abundance of bees). The best model (with the lowest AIC score) in this case was at the 2.5km scale and included the landscape variable percentage built space. Table 3 shows the best GLMs for each of the seven richness and abundance metrics. Altitude, exposure, forb flower abundance, flowering tree abundance, percentage built space, and percentage gardens were all included in some of the best models, although not all of these variables were significant. The models highlighted the following significant patterns: (i) altitude showed a negative relationship with total pollinator richness, (ii) exposure was negatively associated with total pollinator abundance and total richness, (iii) forb flower abundance was positively associated with the richness and abundance metrics, and (iv) percentage built space was negatively associated with the richness and abundance metrics (Table 3). The most consistent pollinator-environment relationships were found for forb flower abundance and percentage built space. The geographical scale of the best model varied with different richness and abundance metrics, but there was some tendency for the bees to be best described at a larger scale (2.5 km) than hoverflies (250 m abundance, 1 km richness). It is worth noting at this point that along an urban-rural gradient many variables co-vary, so the best explanatory variables can potentially act as a proxy for the underlying environmental variable(s) that a species or assemblage is responding to, whether included in the analysis or not. This point is illustrated by Figure 3, which shows co-variability in forb flower abundance and flowering forb species richness, and percentage gardens (at 500 m) and forb flower abundance, for example.

**Table 2:** Generalized linear models for total bee abundance.

Scale	Altitude	Exposure	Forb flower abundance	Built	Gardens	AIC
Site only		0.0561ns	0.0212*			227.4
100m	0.0429*	0.0851ns	0.0105*		0.0249*	225.51
250m		0.0561ns	0.0212*			227.4
500m		0.1061ns		0.0204*		226.87
1km		0.1209ns		0.0111*		225.94
**2.5km**				0.00426**		**225.00**

All models at each geographical scale are shown as an example of the selection process. The 2.5km model was selected as the best model (in bold) based on its lowest akaike information criterion (AIC) value. Probability values of each selected environmental variable are shown (* = *P*<0.05, ** = *P*<0.01, ns = not significant).

**Table 3:** Summary of the selected generalized linear models (GLM) for total bee abundance, total hoverfly abundance, total pollinator abundance, bee species richness, hoverfly species richness, and total pollinator species richness.

Response variable	Scale	GLM model	Altitude	Exposure	Flower ab.	Tree ab.	Built	Gardens
Bee abundance	2.5km	N. binomial					0.0043**	
Hoverfly abundance	250m	N. binomial			0.0007***			0.1138ns
Total abundance	1km	N. binomial		0.0189*	0.0020**		0.0261*	
Bee richness	2.5km	Poisson	0.0522ns	0.1195ns			0.0500*	
Hoverfly richness	1km	Poisson		0.0954ns	0.0111*		0.0069**	
Total richness	2.5km	Poisson	0.0260*	0.0188*	0.0093**	0.0782ns	0.0371*	

The best model for each response variable, according to akaike information criterion values, are shown. Probability values of each selected environmental variable are shown (* = *P*<0.05, ** = *P*<0.01, *** = *P*<0.001, ns  =  not significant). Abbreviations used in the table: N. binomial  =  negative binomial, Flower ab.  =  forb flower abundance, Tree ab.  =  flowering tree abundance.

**Figure 4:**
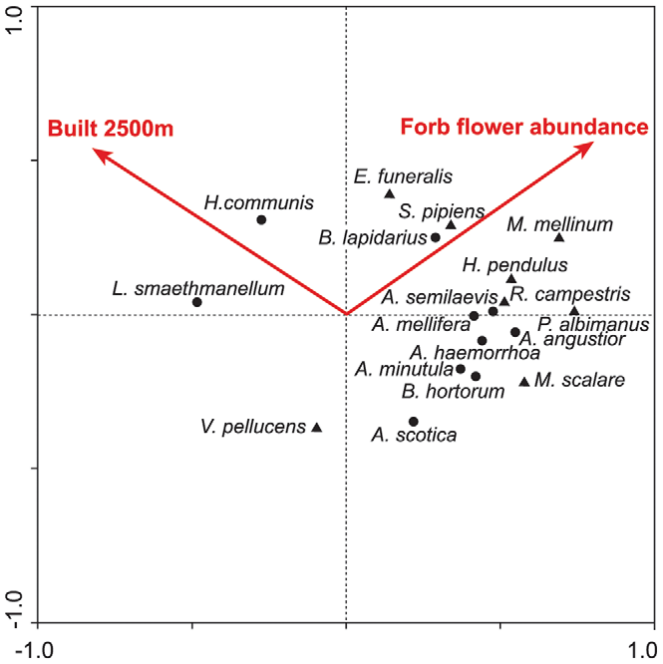
Redundancy analysis (RDA) showing species abundance responses to Built 2500m and Forb flower abundance. The position of bee and hoverfly species in the ordination space are shown with filled circles and triangles respectively. For clarity, only species with the best model fit are illustrated.

**Figure 5:**
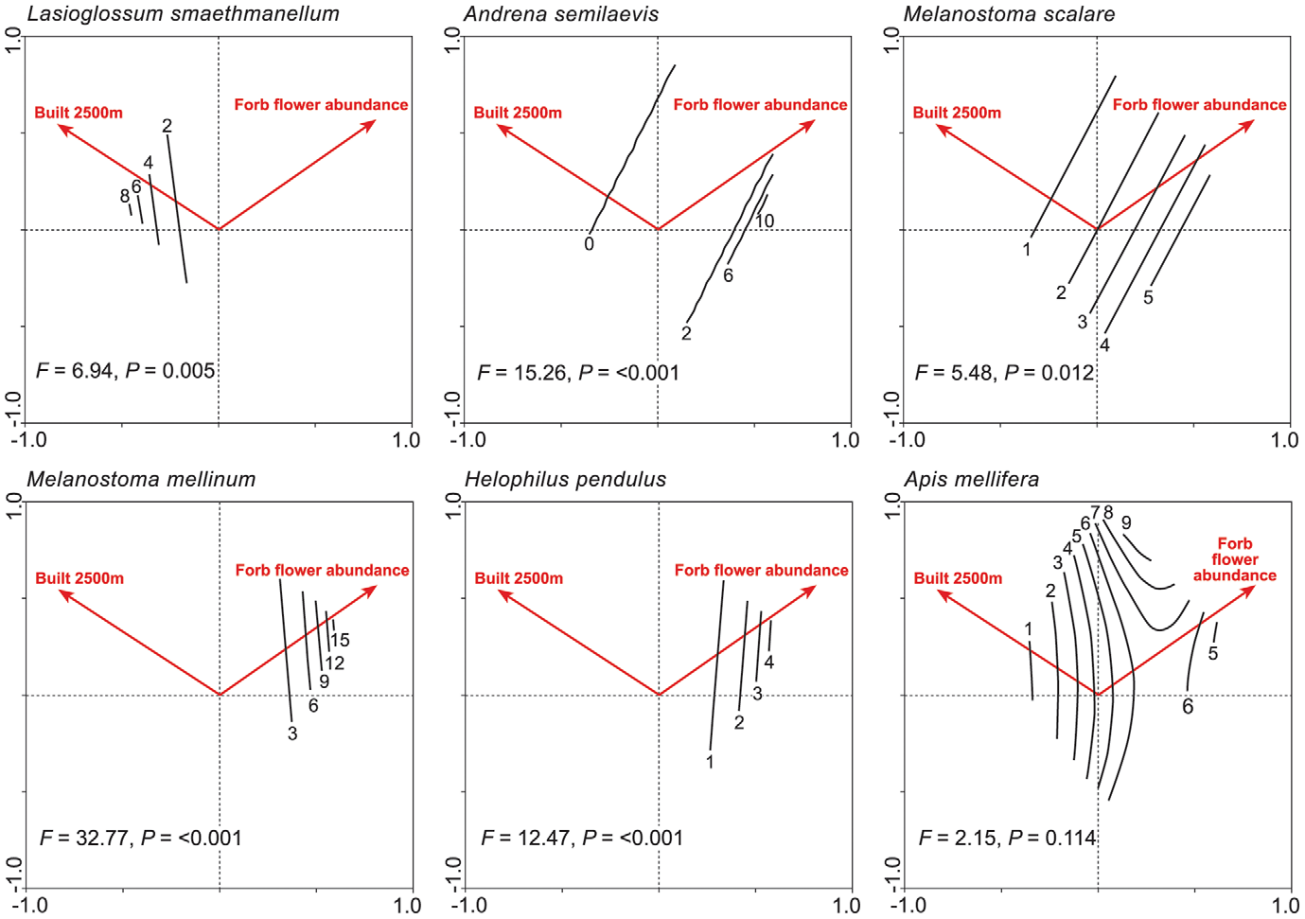
Output from the ordination generalized linear models for six pollinator species. Contours on the plots refer to the predicted abundance values of each species. Vectors are related to the significant explanatory variables.

**Figure 6:**
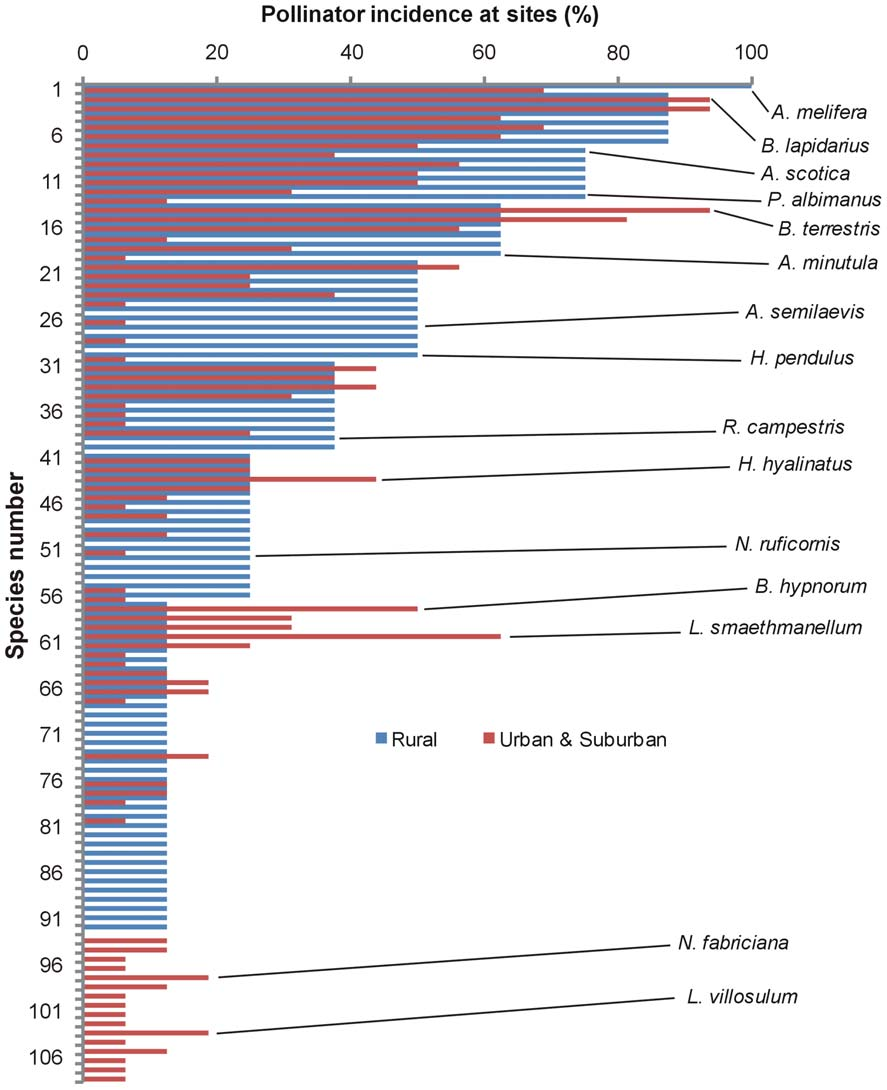
Species incidences of all species at rural, and urban and suburban combined. Data for urban and suburban sites were combined for clarity as they were very similar. Certain species are highlighted, but patterns for all species can be found using the species number in Table S1.

Table 4 shows the ordination statistics for the RDA, which explained 15.3% of the cumulative variance of the species data, and was statistically significant for the first canonical axis. Figure 4 illustrates the results of this RDA in ordination space and shows that percentage built space and forb flower abundance were selected as the variables best describing the total pollinator assemblage at the 2.5 km scale. Some individual species responses are illustrated in Figure 5. Only a few individual species were found to be positively associated with the percentage of built space, and only one species, *Lasioglossum smaethmanellum* was significantly associated with the more urban environment. Several species were found to be negatively associated with percentage built space, with a few of these (e.g. *Andrena semilaevis* and *Melanostoma scalare*) significantly so. Some species were positively associated with forb flower abundance, but often only at sites with low percentage built space (e.g. *Melanostoma mellinum* and *Helophilus pendulus*). Other species (e.g. *Apis mellifera*) did not show significant patterns, but showed a slight preference for more rural sites, with more flower availability. Figure 6 shows a decline in the percentage species incidence at urban and suburban, compared with rural sites. There were few species present at rural sites that were not also present at urban and suburban sites. A few species were more prevalent in urban and suburban sites, including *Bombus terrestris*, *Hylaeus hyalinatus*, *Bombus hypnorum* and *Lasioglossum smaethmanellum*; but many more species were less prevalent at urban and suburban sites such as *Andrena scotica*, *Platycherius albimanus*, *Andrena minutula*, *Andrena semilaevis*, *Helophilus pendulus* and *Rhingia campestris*. Mean abundances of each species at urban, suburban and rural sites are shown in Table S1.

**Table 4:** Eigenvalues, species-environment correlations, cumulative percentage variance in species data explained, and significance of first and all canonical axes in the best redundancy analysis RDA (2.5km scale) for the pollinator species data.

	Axis 1	Axis 2
**Eigenvalues**	0.127	0.026
**Species-environment correlations**	0.723	0.379
**Cumulative% variance of species data**	12.7	15.3
**Significance of first canonical axis**	*F* ratio = 3.055	*P* = 0.0418
**Significance of all canonical axes**	*F* ratio = 1.893	*P* = 0.0520

## Discussion

This study standardized habitat quality as far as possible by sampling the same type of habitat across the urban-rural gradient. However, to some extent habitat quality, in terms of the availability and diversity of flower forage, co-varied along this gradient, with rural sites often characterized by a greater availability and diversity of flower forage than urban and suburban sites. This multicolinearity is inherent in studies of urban-rural gradients, and makes interpretation of results more difficult [36]. Nonetheless, our results show that pollinator assemblages were more diverse and had more individuals in sites with lower levels of urbanization and with the best habitat quality. A negative effect of urbanization was also found in the species data, with more species showing negative relationships with urbanization intensity than positive. We found little difference between assemblages at urban and suburban sites, suggesting that bee and hoverfly assemblages have a threshold response to urbanization, with negative effects established at urbanization levels below that of the ‘suburban’ classification [35] used in this study. Nevertheless, we still sampled diverse pollinator assemblages in urban and suburban areas despite the urbanization effect, particularly where site quality, in terms of the availability of flower forage and more favorable microclimate (lower altitude, less exposure to wind), was good.

The responses of bees to urbanization have varied in other studies, probably in part because of the varied methods, environments and assemblages studied. Nonetheless, some of the findings support the general trends found in our research in Birmingham. Several authors conclude that bee abundance [31], [37], [38] and diversity [37] are most strongly affected by the availability and diversity of flower forage and nesting sites rather than urbanization intensity. Some have also found evidence for a reduction of diversity with urbanization intensity [31], [37], [39]. Some have been encouraged by relatively intact bee assemblages in urban areas [40], [41]. In a study across three cities [42] however, no site quality or landscape variables were found to be consistent predictors of bee assemblage (although flower forage availability was only measured using a management intensity proxy). Other studies have also struggled to find significant overall trends in diversity and abundance [32], [33], and our own Birmingham-based study only explained 15.3% of the variation in the species data in the RDA. Given the potentially wide habitat (*sensu* [43]) area of many species of bees [44] and hoverflies, and the difficulty of adequately assessing habitat quality, especially nesting/overwintering availability [5], in heterogeneous urban landscapes, it is not surprising that some studies have failed to identify consistent environmental determiners of pollinator assemblage structure.

The landscape distribution of a pollinator species is controlled by the distribution of its food, natal, and overwintering resources mediated by its behavior, ecomorphology, and inter-specific interactions [5]. Important factors include: (i) flight ability, which limits dispersal and foraging range in bees and hoverflies, influencing vulnerability to habitat fragmentation [44], [45], [46], (ii) dietary specialization, especially for the larval stage [6], [47], [48], and (iii) nest and overwintering site limitation [38], [49]. These factors are all, to some extent species specific, so pollinator species should differ in their response to urbanization [33], [50], as found for other groups of species [51], [52], [53]. A full analysis of the relationships between species traits and urbanization intensity is outside the scope of this paper, but some initial trends in the species data can be highlighted. Generalist, strong flying, species (e.g. *Apis melifera* and *Bombus lapidarius*) usually demonstrated no negative response to urbanization, whereas more specialist species (e.g. *Helophilus pendulus* larvae require water bodies or damp habitats [54] and *Andrena semilaevis* associated with un-mown/grazed areas of flower rich grassland) are more likely to show a negative response to urbanization. Highly specialized and rare species were infrequent, possibly as a result of the relatively intensive anthropogenic landscape alteration present even in the rural areas. The few species that showed a positive response to urbanization require specialist habitat elements that are more abundant in urbanized areas. For example, *Lasioglossum smaethmanellum* often nests in soft mortar in building walls, and *Bombus hypnorum* tends to specialize on flowers of shrub species usually found in gardens and frequently nests in bird boxes. This was similar to the finding of [33], [50], where cavity nesting specialists were favored by urbanization, presumably because of a greater availability of their nesting resource.

This replacement of some of the pollinator species that decline with urbanization, with species that favor urban habitat elements helps support relatively diverse pollinator assemblages in urban areas. Many bee species and assemblages have been shown to be remarkably resistant to changes in land use, except in cases of extreme habitat loss [8], [16], [55]. Urban areas comprise a highly fragmented mosaic of buildings, built space, parks, gardens, and remnant semi-natural features, which are often small in spatial extent. Each of these elements can potentially provide nesting, overwintering, ovipositing, and foraging partial habitats for bee and hoverfly pollinators, whose flight ability allows the use of multiple habitat elements in their wider habitat (sensu [43]). Urban habitat elements are also subject to a range of disturbance intensities that can support a wide diversity of, often novel, foraging opportunities over long time periods at the wider, whole habitat, scale [3]. Gardens, in particular, have been cited as important habitat elements supporting biodiversity in urban areas [56], [57], [58]. Given the impoverished nature of much agricultural land [7], [59], gardens in urban areas could actually represent increased forage levels for some species, that could help increase pollination levels in surrounding agroecosystems [41], [60], [61]. However, modern gardens are often characterized by a high proportion of horticulturally modified variants of plants and exotic species that can provide poorer quality forage for many pollinators [7], [41], [62]. Gardens have also been shown to provide important nesting opportunities for some species of pollinator [63]. However, we did not detect any positive correlations with percentage area of gardens and any pollinator richness or abundance metric in our study. Furthermore, the treatment with the largest percentage of gardens, suburban, generally had the poorest pollinator faunas.

Sampling in one habitat element does not necessarily give a good indication of highly mobile pollinator assemblage gradient responses across their wider habitat. The landscape context of sampling sites can influence the pollinator assemblage sampled there [5], [64]. In the data-set reported in this paper for example, there may have conceivably been a greater abundance and diversity of pollinators in urban and suburban, than rural treatments (characterized by smaller areas of surrounding garden habitat elements), if most species of pollinator along this gradient use gardens as their main habitat element. Therefore further work on the response of pollinators to urbanization needs to include several types of partial habitat along any urban-rural gradient before definitive findings about the effect of urbanization can be made.

Urbanization effects on insect pollinators may also be expected to vary for different urban areas. Every urban area has a unique geography and development history; therefore it cannot be assumed that the patterns in pollinator assemblages along an urban-rural gradient found for one urban area will necessarily hold for other urban areas. For example, the GLOBENET project [65] sampled carabid beetles along an urban-rural gradient using a common sampling framework across several cities in different countries. It showed that richness and abundance patterns varied widely, with some cities demonstrating a clear decrease in richness and abundance with increasing urbanization, while others did the opposite [20], [51]. Future work on the response of pollinators to urbanization would greatly benefit from a similar standardized approach.

We are not yet at the point where we can draw firm conclusions about the existence, or character, of any universal effects of urbanization on pollinator assemblages. Urban-rural gradients in multiple habitat elements need to be studied in other cities of differing character before this will be possible. Further work will then be needed to carefully document changes in pollinator ecosystem service along these gradients. Nonetheless, results from this and other research suggests that diverse pollinator assemblages can be supported in urban landscapes, particularly in areas of good habitat quality, which is encouraging evidence for the presence of substantial pollination ecosystem services in urban areas.

## Materials and Methods

### Ethics statement

Permission of landowners was obtained for the fieldwork. Permits were not required specifically for the collection of pollinators at the survey sites. However, all efforts were made to engage in ‘collection parsimony’ [66], by only removing pollinators that could not be identified in the field, and using a relatively low intensity of sampling that only removed a sub-set of pollinators at each site.

### Site description and selection

Birmingham (population ∼1 million) is part of the wider West Midlands conurbation (population ∼2.3 million) that includes Solihull, Walsall, Wolverhampton, West Bromwich, Dudley and other parts of the Black Country, that are mainly distributed to the west of The City of Birmingham itself (Figure 6). The conurbation grew rapidly during the industrial revolution, encompassing many surrounding villages and areas of semi-natural habitat, some of which are known to support remnant populations of some species that usually favor more rural habitats around the city [20]. Intensive management since World War II has meant that the UK rural landscape has few areas of semi-natural habitat and unimproved farmland remaining, which has greatly reduced the availability and diversity of pollinator forage and nesting resources [7]. The rural area surrounding Birmingham has fared better than many parts of the UK in terms of the retention of semi-natural and un-cropped areas (e.g. hedgerows) [67].

**Figure 7:**
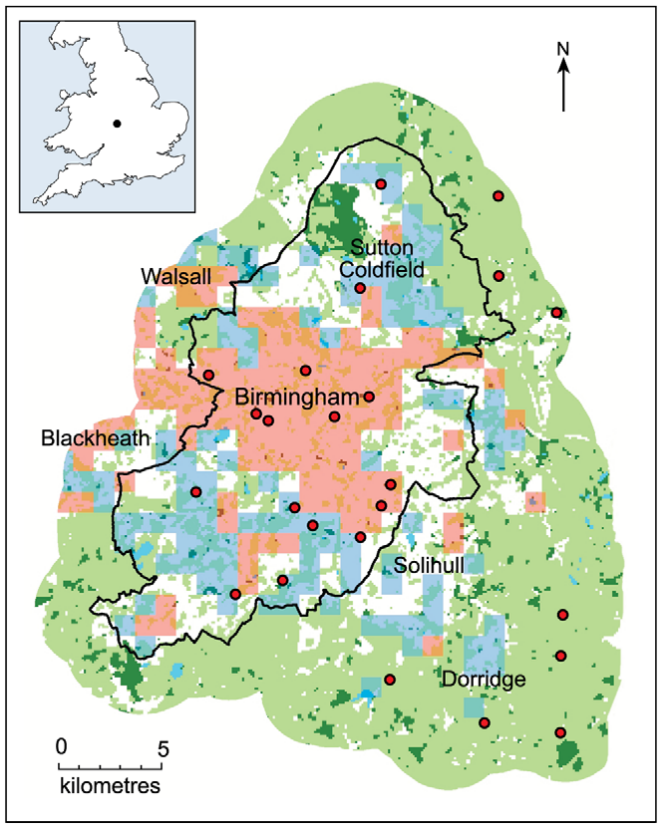
Distribution of the urban, suburban and rural survey sites. Red dots mark the position of each survey site: nine sites in 1km squares classified by Owen et al. (2006) as ‘urban’ or ‘urban transport’ (pink transparency), nine sites in 1km squares classified as ‘suburban’ (blue transparency), and nine rural sites in villages outside Birmingham City limits (black line). Green space is shown as pale green, woodland as dark green, and large still water bodies as blue. Data Crown Copyright/database right 2008 and 2010, an Ordnance Survey/EDINA supplied service.

Sites were selected using the classification of [35], which used principal component and cluster analyses to reduce the dimensionality of data derived from the UK 2000 Land Cover Map, and Ordnance Survey data-sets for the West Midlands conurbation to eight urban classes at a 1 km^2^ scale. Urban sites were selected from within 1 km^2^ squares classified as ‘urban’ or ‘urban transport’; suburban sites were selected from squares with the ‘suburban’ classification, rather than the ‘light suburban’ or ‘dense suburban’ categories; and rural sites were selected from squares with the ‘villages/farms’ category, or from squares outside the classification coverage that had shared characteristics [35]. Few habitat types are both accessible to a rapid sampling regime and consistently encountered at a high enough density throughout urban, suburban and rural land types. Churchyards and cemeteries are one notable exception, which we found could provide reasonably constant habitat character all along the urban-rural gradient and were therefore used for all our sites (Figure 7).

### Pollinator sampling methods

The most efficient and best method for sampling bees over a wide range of geographical regions and habitats is pan trapping [68]. However, some species of bee are poorly sampled using pan trapping [69], and the method performed relatively poorly for hoverflies, so pan trapping was supplemented with sweep netting/hand searching. Three sets (three of each color) of pan traps were used at each sampling site on each sampling occasion. Sets were placed in lightly dappled shade or full sunlight and in a variety of microhabitats across each site to as far as possible maximise the diversity and abundance of catch. Pan traps were made of spray painted (plasti-kote® Projekt Paint _TM_ Gloss Super) plastic takeaway containers (length 16 cm, width10 cm, depth 5 cm) painted white (code 1109), pacific blue (code 1132) and yellow (code 1115), which were half-filled with 3 cm of water that contained a dash of unscented surfactant. Color strongly influences the array of bees and hoverflies caught in pan traps, but yellow, white and blue traps are thought to capture as full a range of species as possible [68], [70], [71], [72]. Traps were used during periods of good weather for bee and hoverfly activity, deemed to be daytime temperatures of 15–25°C with sunny or scattered clouds for spring sampling and 18–25°C with sunny or scattered clouds for summer sampling, and wind speeds <15 km/h.

Traps were installed at half the sites on one day, at the remainder the next day and then retrieved in the same order over the following two days. Hence traps were active for 48 hours, which reduced between site temporal sampling bias (see Table S2). There were five trapping sessions beginning on the following dates: 5/5/09, 23/6/09, 21/7/09, 19/4/10 and 18/5/10. Pan traps are often interfered with by birds (eating invertebrates or soaking bread), ground maintenance staff (strimmed or mown), or members of the public (traps tipped over to rescue invertebrates), and it is not possible to avoid this completely, especially when sites are open access. To account for possible losses we sampled extra sites to begin with so that the sampling design would not be ruined by heavy trap interference. Sites that had a high rate of overall trap interference, or had complete sample interference on any one sampling occasion were removed from the study. In this way the number of sample sites was reduced from 34 to the 24 sites reported here. Average trap returns for urban, suburban and rural sites were 92.4%, 93.8% and 99.3% respectively.

It was not possible to sweep net/hand search simultaneously at each site, so standard samples were taken between 11:00 and 16:00 on days with good weather (see above), to reduce variation due to weather conditions [31], [38]. Low-growing vegetation and trees were swept with a long-handled sweep-net for 30-minutes in each sample. In addition, all bumblebees and *Apis mellifera* that could be identified on the wing were counted during the sweep netting. All distinct microhabitats were investigated at each site. The first sample was taken in late spring 24/5/10 to 7/6/10 and the second in summer 6/7/10 to 30/7/10. Sites were sampled in varying order along the urban to rural gradient in order to limit temporal sampling bias as far as practically possible (see Table S2).

### Local habitat characteristics

Churchyards and cemeteries tend to be highly spatially and temporally heterogeneous in terms of their pollinator forage availability and other habitat characteristics. This makes representative sampling of their local habitat character difficult. Forb flowering plant (forage) species diversity and flower abundance were assessed at each site in May (12/5/10–26/5/10) and July (14/7/10–20/7/10) in approximately 25 m (radius) circles around the centre of sample sites. Forb flower abundance was estimated on a logarithmic scale (10+, 100+ and 1000+) [38], with flower ‘units’ comprising a single flower; or in the case of multi-flowered stems, a single umbel, head, spike or capitulum [73]. The abundance of flowers on insect pollinated tree and woody shrub species is difficult to assess directly because of their height and often short flowering period, but they provide important forage for pollinators. Individual tree and woody shrubs that were able to flower (i.e. not plants in over-cut hedgerows), and at least in part rely on insect pollination for their propagation, were counted in the 25 m radii to provide an index of their forage resource. The exposure of the site to wind was assessed by estimating the total percentage of the horizon within a 50 m (radius) circle not occupied by tall hedgerows, trees or buildings.

### Landscape scale environmental characteristics

Broader scale environmental variation was captured using data in a geographical information system (GIS) in ArcGIS (v.9.3, ESRI Redlands, USA) for Birmingham and the surrounds based on digital layers from the OS Mastermap [74]. Attributes from several polygon fields were grouped to represent broad landcover types and used as the basis for creating a thematic raster with a resolution of 2 m. The following variables were extracted from the GIS: site altitude (m); and the total percentage of: mixed trees, buildings/structures, natural (vegetated) open space, gardens, rivers/canals, stillwater, land under development, roads/paths, manmade open space and rail. The variables buildings/structures, roads/paths, manmade open space, and rail were combined to create a metric of urbanization that was termed ‘built’. Data were extracted using Hawth's Tools [75] at a range of nested spatial scales (100 m, 250 m, 500 m, 1 km and 2.5 km) using concentric circles centered on the sample sites.

### Data analysis

Bee, hoverfly, and total pollinator species richness and abundance metrics were calculated for each site by totalling the different pan trap and sweep netting/hand searching sample counts. Catches of bees and hoverflies are influenced by weather conditions, time of day, date and sampling method. It is important to either minimise this unwanted sampling related variation through careful investigative design, or to include these factors as co-variables in the data analysis. Due to the relatively small number of individuals captured in each sampling event, the use of co-variables was not possible. Instead, temporal variations in sampling efficacy were minimised by sampling in standardised weather conditions and varying the order of sampling (see Pollinator sampling methods above).

Some of the study sites were close enough for the study species to potentially fly between the sites (e.g. [44]), so the data were checked for spatial autocorrelation. Bray-Curtis similarity (sometimes called Sorensen Quantitative Index) values (recommended by [76]) were calculated for pollinator assemblages in all site pairs in EstimateS [77], and the geographical distance between all site pairs were measured. The similarity distance matrix produced was tested for spatial autocorrelation using a Mantel test (1000 permutations in R version 2.12 [78]). No significant spatial autocorrelation was found (*P* = 0.528).

The total abundance and richness metrics were compared using one-way ANOVA when test assumptions of normality and homogeneity of variance were not violated. Frequency histograms and results of Kolmogorov-Smirnoff tests gave no cause for concern regarding the normality of the abundance and richness metrics. However, the between treatment variance for total bee abundance and total abundance were found to be significantly heterogeneous using Levene tests and therefore non-parametric Kruskal-Wallace tests were preferred for these metrics. Tukey and Nemenyi multiple comparison tests were used to identify significant between-treatment differences for ANOVA and Kruskal-Wallace tests respectively [79]. These statistics were calculated using SPSS 15.0.

The relationships between the environmental measures and species richness and abundance metrics were modelled using generalized linear modelling (GLM) in Brodgar v2.6.4 [80] at each of the study scales. Local scale site variables were entered into every model. Many of the GIS-derived environmental variables were found to co-vary at different scales after initial exploration of data using visualisation tools and Pearson's correlations. Colinearity was examined using the variance inflation factors (VIFs) of each explanatory variable entered into the models. Variables with the highest VIF were removed in an iterative process until all VIFs were below 3 [81]. Model validation was done using visualisation tools to check for normality, homogeneity and independence [81]. The deviance/degrees of freedom ratio was used to assess possible over-dispersion in the models [81]. Poisson distributions were most appropriate for species richness metrics, and negative binomial distributions were used for abundance metrics, which were over-dispersed [82]. Models were selected using akaike information criterion (AIC) [83] for all study spatial scales.

Individual species responses to environmental explanatory environmental gradients were assessed using ordination in Canoco for Windows version 4.51 [84]. The gradient lengths from initial indirect ordinations using detrended correspondence analysis (DCA) were all short (<3) so redundancy analysis (RDA) was selected as the most appropriate ordination method [85]. Scaling focused on inter-species correlations and species scores divided by their standard deviation were used for RDAs. Model significance values were generated using Monte Carlo analyses (9999 permutations, with a random seed). Models were constructed for each geographical scale, with only individually significant environmental variables used in the final model. The final selected model explained the highest cumulative percentage of species data. Individual species responses to environmental gradients in the ordination space were assessed using GLM species data attribute plots with Poisson distributions and the best linear or quadratic fit depending on AIC score.

## Supporting Information

Table S1 **Mean number of each species at urban, suburban and rural sites.** Species are arranged in order of decreasing site incidence under the rural treatment to link with Figure 6.
(DOC)

Table S2 **Order and dates that sites were visited for pan trapping and hand searching/sweep netting.**
(DOC)
