# Supplementary material for: Changing Bee and Hoverfly Pollinator Assemblages along an Urban-Rural Gradient
Source: PLoS One. 2011 Aug 12;6(8):e23459. doi: 10.1371/journal.pone.0023459 (PMC3155562; doi:10.1371/journal.pone.0023459)
Supplement: Table S1 — Mean number of each species at urban, suburban and rural sites. Species are arranged in order of decreasing site incidence under the rural treatment to link with Figure 6. (DOC) [file pone.0023459.s001.doc]

|  | **Species Number** | **Urban** | **Suburban** | **Rural** |
| --- | --- | --- | --- | --- |
| *Apis melifera* Linnaeus, 1758 | 1 | 3.13 | 2.38 | 6.25 |
| *Bombus lapidarius* (Linnaeus, 1758) | 2 | 3.25 | 6.38 | 5.13 |
| *Bombus pascuorum* (Scopoli, 1763) | 3 | 3.13 | 2.38 | 2.88 |
| *Melanostoma scalare* (Fabricius, 1794) | 4 | 0.50 | 3.00 | 4.00 |
| *Episyrphus balteatus* (De Geer, 1776) | 5 | 5.38 | 12.38 | 10.13 |
| *Syritta pipiens* (Linnaeus, 1758) | 6 | 2.50 | 2.25 | 3.88 |
| *Bombus vestalis* (Geoffroy in Fourcroy, 1785) | 7 | 1.63 | 1.00 | 2.75 |
| *Andrena scotica* Perkins, R.C.L., 1916 | 8 | 0.75 | 0.63 | 2.63 |
| *Andrena bicolor* Fabricius, 1775 | 9 | 2.13 | 0.75 | 2.13 |
| *Andrena haemorrhoa* (Fabricius, 1781) | 10 | 1.25 | 0.75 | 14.13 |
| *Osmia rufa* (Linnaeus, 1758) | 11 | 2.50 | 1.75 | 7.75 |
| *Bombus hortorum* (Linnaeus, 1761) | 12 | 0.38 | 0.38 | 1.75 |
| *Platycherius albimanus* (Fabricius, 1781) | 13 | 0.13 | 0.13 | 1.63 |
| *Bombus terrestris* (Linnaeus, 1758) | 14 | 2.50 | 2.63 | 1.00 |
| *Bombus pratorum* (Linnaeus, 1761) | 15 | 3.88 | 3.75 | 2.38 |
| *Bombus lucorum* (Linnaeus, 1761) | 16 | 0.63 | 1.75 | 2.13 |
| *Melanostoma mellinum* (Linnaeus, 1758) | 17 | 0.13 | 0.13 | 5.25 |
| *Merodon equestris* (Fabricius, 1794) | 18 | 0.50 | 0.13 | 1.13 |
| *Andrena minutula* (Kirby, 1802) | 19 | 0.25 | 0.00 | 1.63 |
| *Platycheirus scutatus* agg. | 20 | 2.50 | 0.63 | 1.13 |
| *Nomada flava* Panzer, 1798 | 21 | 0.63 | 0.25 | 2.00 |
| *Eumerus funeralis* Meigen, 1822 | 22 | 1.50 | 2.13 | 1.25 |
| *Lasioglossum calceatum* (Scopoli, 1763) | 23 | 1.75 | 0.13 | 2.38 |
| *Cheilosia albitarsis* (Meigen, 1822) | 24 | 0.00 | 0.13 | 1.25 |
| *Andrena angustior* (Kirby, 1802) | 25 | 0.00 | 0.00 | 1.75 |
| *Andrena cineraria* (Linnaeus, 1758) | 26 | 0.13 | 0.00 | 2.38 |
| *Andrena semilaevis* Pérez, 1903 | 27 | 0.00 | 0.00 | 3.75 |
| *Nomada panzeri* Lepeletier, 1841 | 28 | 0.38 | 0.00 | 0.75 |
| *Cheilosia soror* (Zetterstedt, 1843) | 29 | 0.00 | 0.00 | 0.50 |
| *Helophilus pendulus* (Linnaeus, 1758) | 30 | 0.13 | 0.00 | 2.00 |
| *Bombus sylvestris* (Lepeletier, 1832) | 31 | 1.38 | 1.38 | 0.63 |
| *Syrphus ribesii* (Linnaeus, 1758) | 32 | 0.38 | 0.88 | 0.63 |
| *Andrena fulva* (Müller in Allioni, 1776) | 33 | 2.38 | 1.13 | 1.00 |
| *Lasioglossum albipes* (Fabricius, 1781) | 34 | 0.25 | 0.75 | 1.63 |
| *Andrena nigroaenea* (Kirby, 1802) | 35 | 0.00 | 0.13 | 0.75 |
| *Nomada goodeniana* (Kirby, 1802) | 36 | 0.00 | 0.13 | 1.00 |
| *Platycherius clypteatus* (Meigen, 1822) | 37 | 0.00 | 0.13 | 1.25 |
| *Hylaeus communis* Nylander, 1852 | 38 | 0.88 | 0.00 | 0.38 |
| *Rhingia campestris* Meigen, 1822 | 39 | 0.00 | 0.00 | 2.88 |
| *Neoascia podagrica* (Fabricius, 1775) | 40 | 0.00 | 0.00 | 1.88 |
| *Dasysyrphus albostriatus* (Fallén, 1817) | 41 | 0.13 | 0.38 | 0.75 |
| *Volucella pellucens* (Linnaeus, 1758) | 42 | 0.13 | 0.88 | 0.25 |
| *Hylaeus hyalinatus* Smith, F., 1842 | 43 | 4.50 | 1.13 | 0.38 |
| *Andrena nitida* (Müller, 1776) | 44 | 1.13 | 0.13 | 2.13 |
| *Megachile centuncularis* (Linnaeus, 1758) | 45 | 0.13 | 0.13 | 0.25 |
| *Bombus barbutellus* (Kirby, 1802) | 46 | 0.00 | 0.13 | 0.25 |
| *Bombus rupestris* (Fabricius, 1793) | 47 | 0.13 | 0.13 | 0.75 |
| *Andrena subopaca* Nylander, 1848 | 48 | 0.00 | 0.00 | 0.38 |
| *Halictus tumulorum* (Linnaeus, 1758) | 49 | 0.38 | 0.00 | 0.25 |
| *Megachile ligniseca* (Kirby, 1802) | 50 | 0.00 | 0.00 | 0.25 |
| *Nomada flavoguttata* (Kirby, 1802) | 51 | 0.13 | 0.00 | 0.38 |
| *Nomada ruficornis* (Linnaeus, 1758) | 52 | 0.00 | 0.00 | 0.25 |
| *Platycherius granditarsis* (Forster, 1771) | 53 | 0.00 | 0.00 | 0.25 |
| *Chrysogaster solstitialis* (Fallén, 1817) | 54 | 0.00 | 0.00 | 0.25 |
| *Melanogaster hirtella* (Loew, 1843) | 55 | 0.13 | 0.00 | 0.25 |
| *Xylota segnis* (Linnaeus, 1758) | 56 | 0.13 | 0.00 | 2.13 |
| *Bombus hypnorum* (Linnaeus, 1758) | 57 | 1.00 | 1.75 | 0.25 |
| *Eupeodes corollae* (Fabricius, 1794) | 58 | 0.13 | 0.63 | 0.25 |
| *Eupeodes latifasciatus* (Macquart, 1829) | 59 | 0.25 | 1.00 | 0.13 |
| *Lasioglossum smeathmanellum* (Kirby, 1802) | 60 | 4.25 | 0.63 | 0.13 |
| *Epistrophe eligans* (Harris, 1780) | 61 | 0.50 | 0.25 | 0.13 |
| *Colletes daviesanus* Smith, F., 1846 | 62 | 0.00 | 0.13 | 0.13 |
| *Bombus campestris* (Panzer, 1801) | 63 | 0.00 | 0.13 | 0.13 |
| *Eupeodes luniger* (Meigen, 1822) | 64 | 0.13 | 0.13 | 0.13 |
| *Syrphus vitripennis* Meigen, 1822 | 65 | 0.25 | 0.13 | 0.13 |
| *Eristalis pertinax* (Scopoli, 1763) | 66 | 0.25 | 0.13 | 0.13 |
| *Eristalis tenax* (Linnaeus, 1758) | 67 | 0.00 | 0.13 | 0.13 |
| *Andrena helvola* (Linnaeus, 1758) | 68 | 0.00 | 0.00 | 0.88 |
| *Andrena synadelpha* Perkins, R.C.L., 1914 | 69 | 0.00 | 0.00 | 0.38 |
| *Andrena chrysosceles* (Kirby, 1802) | 70 | 0.00 | 0.00 | 0.25 |
| *Andrena dorsata* (Kirby, 1802) | 71 | 0.00 | 0.00 | 0.13 |
| *Andrena wilkella* (Kirby, 1802) | 72 | 0.00 | 0.00 | 0.50 |
| *Lasioglossum leucopus* (Kirby, 1802) | 73 | 1.38 | 0.00 | 0.13 |
| *Sphecodes ferruginatus* von Hagens, 1882 | 74 | 0.00 | 0.00 | 0.13 |
| *Chelostoma florisomne* (Linnaeus, 1758) | 75 | 0.00 | 0.00 | 0.25 |
| *Osmia caerulescens* (Linnaeus, 1758) | 76 | 0.25 | 0.00 | 0.38 |
| *Osmia leaiana* (Kirby, 1802) | 77 | 0.25 | 0.00 | 0.13 |
| *Nomada marshamella* (Kirby, 1802) | 78 | 0.25 | 0.00 | 0.13 |
| *Bombus ruderatus* (Fabricius, 1775) | 79 | 0.00 | 0.00 | 0.13 |
| *Dasysyrphus venustus* (Meigen, 1822) | 80 | 0.25 | 0.00 | 0.25 |
| *Xanthogramma pedissequum* (Harris, 1776) | 81 | 0.00 | 0.00 | 0.13 |
| *Cheilosia antiqua* (Meigen, 1822) | 82 | 0.00 | 0.00 | 0.13 |
| *Cheilosia illustrata* (Harris, 1780) | 83 | 0.00 | 0.00 | 0.13 |
| *Cheilosia impressa* Loew, 1840 | 84 | 0.00 | 0.00 | 0.13 |
| *Cheilosia lasiopa* Kowarz, 1885 | 85 | 0.00 | 0.00 | 0.13 |
| *Ferdinandea cuprea* (Scopoli, 1763) | 86 | 0.00 | 0.00 | 0.13 |
| *Portevinia maculata* (Fallén, 1817) | 87 | 0.00 | 0.00 | 0.13 |
| *Riponnensia splendens* (Meigen, 1822) | 88 | 0.00 | 0.00 | 0.13 |
| *Eristalis arbustorum* (Linnaeus, 1758) | 89 | 0.00 | 0.00 | 0.13 |
| *Helophilus hybridus* Loew, 1846 | 90 | 0.00 | 0.00 | 0.13 |
| *Chalcosyrphus nemorum* (Fabricius, 1805) | 91 | 0.00 | 0.00 | 0.13 |
| *Xylota tarda* Meigen, 1822 | 92 | 0.00 | 0.00 | 0.13 |
| *Parasyrphus nigritarsis* (Zetterstedt, 1843) | 93 | 0.00 | 0.25 | 0.00 |
| *Halictus rubicundus* (Christ, 1791) | 94 | 0.25 | 0.13 | 0.00 |
| *Sphecodes ephippius* (Linnaeus, 1767) | 95 | 0.00 | 0.13 | 0.00 |
| *Coelioxys inermis* (Kirby, 1802) | 96 | 0.00 | 0.13 | 0.00 |
| *Nomada fabriciana* (Linnaeus, 1767) | 97 | 0.25 | 0.13 | 0.00 |
| *Baccha elongata* (Fabricius, 1775) | 98 | 0.13 | 0.13 | 0.00 |
| *Melangyna umbellatarum* (Fabricius, 1794) | 99 | 0.00 | 0.13 | 0.00 |
| *Scaeva pyrastri* (Linnaeus, 1758) | 100 | 0.00 | 0.50 | 0.00 |
| *Andrena denticulata* (Kirby, 1802) | 101 | 0.13 | 0.00 | 0.00 |
| *Melitta haemorrhoidalis* (Fabricius, 1775) | 102 | 0.13 | 0.00 | 0.00 |
| *Lasioglossum villosulum* (Kirby, 1802) | 103 | 0.63 | 0.00 | 0.00 |
| *Meliscaeva auricollis* (Meigen, 1822) | 104 | 0.13 | 0.00 | 0.00 |
| *Sphaerophoria scripta* (Linnaeus, 1758) | 105 | 0.88 | 0.00 | 0.00 |
| *Myathropa florea* (Linnaeus, 1758) | 106 | 0.13 | 0.00 | 0.00 |
| *Heringia brevidens* (Egger, 1865) | 107 | 0.13 | 0.00 | 0.00 |
| *Volucella inanis* (Linnaeus, 1758) | 108 | 0.13 | 0.00 | 0.00 |
