# Supplementary material for: Changing Bee and Hoverfly Pollinator Assemblages along an Urban-Rural Gradient
Source: PLoS One. 2011 Aug 12;6(8):e23459. doi: 10.1371/journal.pone.0023459 (PMC3155562; doi:10.1371/journal.pone.0023459)
Supplement: Table S2 — Order and dates that sites were visited for pan trapping and hand searching/sweep netting. (DOC) [file pone.0023459.s002.doc]

| **Site name** | **Type** | **Pan trapping order & days sampled** | **Hand search 1** | **Hand search 2** |
| --- | --- | --- | --- | --- |
| St Nicolas, Kings Norton | Suburban | 1 (day 1 & 3) | 24/05/2010 | 06/07/2010 |
| St Peter, Harborne | Suburban | 2 (day 1 & 3) | 04/06/2010 | 26/07/2010 |
| St Paul, Birmingham | Urban | 3 (day 1 & 3) | 28/05/2010 | 29/07/2010 |
| Warstone Lane Cemetery | Urban | 4 (day 1 & 3) | 28/05/2010 | 08/07/2010 |
| St James, Handsworth | Urban | 5 (day 1 & 3) | 28/05/2010 | 08/07/2010 |
| SS Peter & Paul, Aston & Nechells | Urban | 6 (day 1 & 3) | 28/05/2010 | 08/07/2010 |
| St Saviour, Saltley | Urban | 7 (day 1 & 3) | 07/06/2010 | 29/07/2010 |
| St Margaret, Ward End | Urban | 8 (day 1 & 3) | 28/05/2010 | 29/07/2010 |
| St Michael, Boldmere | Suburban | 9 (day 1 & 3) | 28/05/2010 | 19/07/2010 |
| St James, Hill | Suburban | 10 (day 1 & 3) | 02/06/2010 | 19/07/2010 |
| St John the Baptist, Middleton | Rural | 11 (day 1 & 3) | 02/06/2010 | 19/07/2010 |
| St Chad, Wishaw | Rural | 12 (day 1 & 3) | 02/06/2010 | 19/07/2010 |
| St John the Baptist, Lea Marston | Rural | 13 (day 1 & 3) | 02/06/2010 | 19/07/2010 |
| St Swithin, Barston | Rural | 14 (day 1 & 3) | 25/05/2010 | 21/07/2010 |
| St Mary the Virgin, Temple Balsall | Rural | 15 (day 1 & 3) | 25/05/2010 | 21/07/2010 |
| St Michael, Baddesley Clinton | Rural | 16 (day 1 & 3) | 25/05/2010 | 21/07/2010 |
| St Giles, Packwood | Rural | 17 (day 2 & 4) | 25/05/2010 | 21/07/2010 |
| St Patrick's, Earlswood | Rural | 18 (day 2 & 4) | 25/05/2010 | 21/07/2010 |
| Yardley Cemetery | Urban | 19 (day 2 & 4) | 04/06/2010 | 06/07/2010 |
| St Mary, Acocks Green | Urban | 20 (day 2 & 4) | 04/06/2010 | 06/07/2010 |
| Church of the Ascension, Hall Green | Suburban | 21 (day 2 & 4) | 04/06/2010 | 06/07/2010 |
| St Agnes, Moseley | Suburban | 22 (day 2 & 4) | 24/05/2010 | 30/07/2010 |
| St Mary, Moseley | Suburban | 23 (day 2 & 4) | 24/05/2010 | 30/07/2010 |
| Brandwood End Cemetery | Suburban | 24 (day 2 & 4) | 24/05/2010 | 06/07/2010 |
